# SPAD array chips with full frame readout for crystal characterization

**DOI:** 10.1186/2197-7364-2-S1-A3

**Published:** 2015-05-18

**Authors:** Peter Fischer, Roberto Blanco, Ilaria Sacco, Michael Ritzert, Sascha Weyers

**Affiliations:** Heidelberg University, Germany; Fraunhofer Institute for Microelectronic Circuits and Systems, Germany

We present single photon sensitive 2D camera chips containing 88x88 avalanche photo diodes which can be read out in full frame mode with up to 400.000 frames per second. The sensors have an imaging area of ~5mm x 5mm covered by square pixels of ~56µm x 56µm with a ~55% fill factor in the latest chip generation. The chips contain a self triggering logic with selectable (column) multiplicities of up to >=4 hits within an adjustable coincidence time window. The photon accumulation time window is programmable as well. First prototypes have demonstrated low dark count rates of <50kHz/mm2 (SPAD area) at 10 degree C for 10% masked pixels. One chip version contains an automated readout of the photon cluster position. The readout of the detailed photon distribution for single events allows the characterization of light sharing, optical crosstalk etc., in crystals or crystal arrays as they are used in PET instrumentation. This knowledge could lead to improvements in spatial or temporal resolution.

